# How Does Teacher Certification Promote Student Achievement in Science, Reading, and Math? A Chain-Mediated Model of Teachers’ Sense of Efficacy and Pedagogical Innovation

**DOI:** 10.3390/jintelligence14010002

**Published:** 2025-12-22

**Authors:** Yanbin Guo, Guoxiu Tian

**Affiliations:** College of Teacher Education, Capital Normal University, Beijing 100048, China

**Keywords:** teacher certification, student achievement, teachers’ sense of efficacy, pedagogical innovation, Türkiye

## Abstract

Teacher certification is strongly correlated with student development. Many studies have documented the effect of teacher certification on student achievement. However, there are inconsistent conclusions about this issue. Moreover, few studies have examined the mechanisms by which teacher certification promotes student achievement. To fill these gaps, this paper examines the effect of teacher certification on student achievement and the underlying mechanisms. We analyzed the data from the TALIS 2018 Türkiye teacher data and the PISA 2018 Türkiye student data using path analysis and PROCESS Model 6. It was found that the rise in entry requirements for teacher certification was positively associated with teachers’ sense of efficacy and pedagogical innovation in the Turkish context. It was also indicated that teacher certification was positively associated with student achievement through the serial mediation of teachers’ sense of efficacy and pedagogical innovation. The practical and theoretical implications of this paper were discussed.

## 1. Introduction

Although social-emotional skills have attracted much attention in recent years, student cognitive skills (e.g., student achievement) are still crucial for school effectiveness (Danielson, 2002), labor market outcomes (Ge et al., 2022), and life wellbeing (Clark & Del Bono, 2016). Student achievement is affected by school financial factors (Jackson & Mackevicius, 2024), student-related factors (Dunlosky et al., 2020), parental factors (Doepke et al., 2019), and teacher characteristics (Coenen et al., 2018). In addition, in recent years, teacher characteristics, especially teacher certification, have received increasing academic attention (Chung & Zou, 2025; Guo, 2024; Larsen et al., 2020; Nawas et al., 2025). As one of the occupational licenses, teacher certification aims to identify and select qualified and quality teachers for basic education schools (Goldhaber, 2011). The thresholds for teacher certification are strongly correlated with student outcomes (Gordon et al., 2023). Some studies have documented that teacher certification can positively predict student achievement (K. A. Anderson, 2020; Nawas et al., 2025). However, another strand of literature has found no significant correlation between teacher certification and student achievement (Coenen et al., 2018). Moreover, few studies have explored the mechanisms by which teacher certification promotes student achievement (K. A. Anderson, 2020). Considering the complexity of factors that shape student achievement, understanding how teacher certification promotes it is crucial for designing targeted policies that can lead to improvement.

Teacher certification, an occupational license in the education field (Goldhaber, 2011), enhances teacher outcomes through several key mediators. Teachers’ sense of efficacy and pedagogical innovation play crucial roles in the mechanisms through which teacher certification promotes student achievement. On one hand, teachers’ sense of efficacy is a subjective feeling regarding their own knowledge, skills, and abilities (Gordon et al., 2023). After thresholds for teacher certification rise, the number of candidates who ultimately obtain teacher certification undoubtedly declines. Thus, these successful candidates tend to believe in their own abilities and develop higher self-efficacy. On the other hand, pedagogical innovation is a series of creative activities that heavily depend on teachers’ ability and level (S. Liu et al., 2024). Teachers who excel in pedagogical innovation are typically highly capable in both pedagogy theory and practices, and they possess independent pedagogical thinking, which is characterized by traits like a strong sense of efficacy—a key factor in effectively improving student achievement. High thresholds for teacher certification facilitate selecting highly innovative teachers. Thus, teacher certification is positively correlated with pedagogical innovation. Additionally, many studies have confirmed the positive relationship between teachers’ sense of efficacy and student achievement (Y. Liu et al., 2022) and between pedagogical innovation and student achievement (Gil-Izquierdo et al., 2023). Thus, teachers’ sense of efficacy and pedagogical innovation may play mediating roles in the relationship between teacher certification and student achievement.

This paper analyzed the TALIS 2018 Türkiye teacher data and the PISA 2018 Türkiye student data using path analysis and PROCESS Model 6. Grounded in public interest theory (PIT) and Bandura’s self-efficacy theory, this study examined the mediating effect of teachers’ sense of efficacy and pedagogical innovation in this relationship between teacher certification and student achievement. Specifically, the following research questions are proposed:

RQ1: Was teacher certification positively associated with student achievement?

RQ2: Do teachers’ sense of efficacy and pedagogical innovation mediate the relationship between teacher certification and student achievement?

In addition, the hypotheses are proposed:

**H1.**  *Teacher certification is significantly and positively related to student achievement.*

**H2a.**  *Teacher certification is positively and significantly related to teachers’ sense of efficacy.*

**H2b.**  *Teachers’ sense of efficacy is positively and significantly related to student achievement.*

**H3a.**  *Teacher certification is positively and significantly correlated with pedagogical innovation.*

**H3b.**  *Pedagogical innovation is positively and significantly correlated with student achievement.*

**H4a.**  *Teachers’ sense of efficacy is positively and significantly associated with pedagogical innovation.*

**H4b.**  *Teachers’ sense of efficacy and pedagogical innovation serve as chain mediators in the relationship between teacher certification and student achievement.*

## 2. Literature Review and Hypothesis

### 2.1. Conceptual Framework

This study was guided by PIT and Bandura’s self-efficacy theory. The first one is PIT. Like lawyers’, drivers’, medical practitioners’ licenses, and other occupational licenses, teacher certification is a form of occupational licensing (Goldhaber, 2011). Occupational licensing refers to the licensure required to legally practice a certain occupation (M. Kleiner, 2000). The PIT is a prominent theory for explaining the effects of occupational licensing (Maurizi, 1974). The theory posits that occupational licensing screens out unqualified and low-quality candidates by setting a high threshold (e.g., exam passing scores) and thereby promotes the average quality of the product or service (S. Liu et al., 2024). This theoretical prediction has been confirmed in many occupations: pilots (Rupp & Tan, 2025), nurses (D. M. Anderson et al., 2020), medical doctors (M. M. Kleiner, 2015), and teachers (Goldhaber et al., 2025). From this theoretical perspective, as a form of occupational licensure, teacher certification is crucial for selecting qualified and quality candidates. Teacher quality is strongly associated with thresholds for teacher certification. Different types of teacher certification have different entry requirements. Typically, thresholds for teacher certification become progressively lower from traditional certification and alternative certification to uncertified certification. There are many measures to assess teacher quality. Student achievement is one of the most widely recognized indictors for assessing teacher quality (Hanushek & Rivkin, 2006). Thus, whether raising teacher certification thresholds improves student achievement is a key question for evaluating the impact of such a policy.

The second theory is Bandura’s self-efficacy theory (Bandura & Adams, 1977). It posits that individual perceived self-efficacy shapes their behaviors and decisions. When facing a task, an individual typically evaluates its value. However, not all valuable tasks are accepted. Individuals typically assess their abilities and make decisions. Individuals with high self-efficacy actively embrace and receive valuable tasks. Moreover, through their practical experiences, individuals refine their sense of efficacy as well as their goals and approaches based on the feedback received. The theory has been widely used in the study of teacher behavior (Chen et al., 2020; Uysal, 2025). In summary, PIT and Bandura’s self-efficacy theory provide a conceptual grounding for our study.

### 2.2. Teacher Certification and Student Achievement

Teacher certification is defined as an occupational license that teachers obtain after meeting a series of requirements (Goldhaber, 2011). Student achievement in this paper refers to students’ performance in math, science, and reading. According to the PIT, stringent occupational licensing can improve the average quality of products or services (Maurizi, 1974). Students are the most important “product” of the educational process, and teachers are practitioners who develop these “products” through school education. Moreover, student achievement is both one of the most important indicators and a direct reflection of teacher quality, and it is also the most convincing measure for assessing the effect of variations in teacher certification thresholds. Specifically, taking written exams on subject and pedagogical knowledge is one of the common methods for obtaining teacher certification. Raising the thresholds for teacher certification typically raises the passing score on these exams. Thus, when thresholds for teacher certification rise, the candidates who pass these written exams are likely to be those who are good at taking exams (Goldhaber, 2011). Therefore, this screening process undoubtedly contributes to improving student achievement. In this regard, teacher certification can positively predict student achievement. In addition, based on 1319 Indonesian secondary schools, one study found that teacher certification positively predicted student achievement through job satisfaction, collaboration, and student learning motivation (Nawas et al., 2025). Another study found that teacher certification was positively related to student achievement (e.g., middle school math achievement). However, these effects varied by subject, grade level, and certification types (Cowan & Goldhaber, 2016). Compared to alternative teacher certifications, traditional teacher certification showed a stronger positive correlation with student achievement in a sample of U.S. high schools (K. A. Anderson, 2020). However, a further study also found no link between teacher certification and student achievement (Chung & Zou, 2025).

### 2.3. The Mediation of Teachers’ Sense of Efficacy

Teachers’ sense of efficacy refers to their beliefs about their capacity to succeed in teaching and education (Teng et al., 2024). According to the PIT, thresholds for teacher certification are strongly correlated with the level of teachers’ professional skills and abilities (Goldhaber, 2011). As thresholds for teacher certification rise, the requirements for teacher comprehensive competencies also rise. Teachers with high competencies usually have strong confidence in their ability to handle their teaching tasks. Given that the sense of efficacy is a subjective judgement of one’s own competence, the level of teachers’ sense of efficacy can improve the quality of their teaching and students’ enthusiasm for learning (Schiefele, 2017). These two aspects are strongly correlated with student achievement. Specifically, teachers with a high sense of efficacy have their own unique and effective pedagogical skills and philosophies, which in turn lead to higher student achievement. Meanwhile, teachers with a high sense of efficacy are good at helping students develop their interests in learning, which in turn enhances their intrinsic motivation to learn and further improves their achievement. In addition, based on the TALIS2018 Taiwan data, one study found that teachers with a high sense of efficacy contributed to improving pedagogical practices (Chen et al., 2020), an improvement which was linked to higher student achievement. Using data from China, another study found that a high sense of efficacy among teachers could promote student achievement (Y. Liu et al., 2022). Using a two-level meta-analysis, a further study found that teachers’ sense of efficacy was positively related to student achievement (Li & Liu, 2022). Using a sample of 1319 secondary school teachers in Indonesia, a subsequent study found that teachers’ sense of efficacy was positively and directly associated with student achievement (Nawas et al., 2025).

### 2.4. The Mediation of Pedagogical Innovation

Pedagogical innovation is defined as a deliberate process encompassing idea generation, development, implementation, evaluation, modification, dissemination, continuation, and the realization of anticipated benefits (S. Liu et al., 2024). From the PIT perspective (Maurizi, 1974), teacher certification aims to attract and select qualified and quality teaching candidates through rigorous screening. After thresholds for teacher certification rise, those who can obtain teacher certification are more likely to be highly competent individuals. These teachers not only excel at their teaching duties but also have the ambition and extra time to engage in pedagogical innovation. Specifically, teachers with high competencies typically have high career ambitions and clear career plans. Put differently, driven by long-term and ambitious career plans, they are sufficiently motivated to actively improve their pedagogical strategies (Jang, 2019), which in turn enhances student learning. Meanwhile, teachers with high competencies usually complete their teaching tasks and other duties assigned by administrators quickly. Thus, they have more time to explore new pedagogical methods and acquire cutting-edge pedagogical knowledge. In addition, teachers with high competencies are more likely to build positive teacher–student relationships, which in turn contribute to experimenting with new pedagogical strategies. Overall, high thresholds for teacher certification facilitate the selection of innovative teachers. In addition, using the propensity score method, one study found that, compared with traditional teaching strategies, innovative pedagogical practices were positively correlated with students’ reading achievement (Gil-Izquierdo et al., 2023). Another study found that pedagogical innovation can positively predict student achievement in computer programming (Omeh & Olelewe, 2021).

### 2.5. The Chain-Mediating Role Between Teachers’ Sense of Efficacy and Pedagogical Innovation

From the perspective of the PIT, a higher threshold for teacher certification contributes to selecting qualified and quality candidates (Goldhaber, 2011). On one hand, qualified and quality teachers are much more likely to be those who do well in teaching and educational work (Nawas et al., 2025). They are typically good at building positive teacher–student relationships, developing innovative pedagogical methods, and improving student achievement (Liao et al., 2024). These advantages give them strong confidence and a high sense of efficacy in their teaching and educational abilities (Burić et al., 2024). On the other hand, according to Bandura’s self-efficacy theory, individuals’ self-efficacy affects their behavior and decisions (Bandura & Adams, 1977). The relationship between teachers’ sense of efficacy and pedagogical innovation can be understood through the lens of this theory. Specifically, teachers with a high sense of efficacy are typically more likely to explore new pedagogical strategies and develop pedagogical philosophies. Combined, when thresholds for teacher certification are raised, the screening process is much more likely to identify individuals with a high sense of efficacy and a talent for pedagogical innovation. In addition, a lot of studies have indicated that teachers’ sense of efficacy was positively associated with pedagogical innovation. For example, based on a sample of 41 primary and high schools in China, one study found that teachers’ sense of efficacy could promote pedagogical innovation (Cai & Tang, 2021). Using TALIS 2018 Taiwan teacher data, another study found that teachers’ sense of efficacy could positively predict pedagogical innovation (Hsieh et al., 2024). Using structural equation models, a further study found that teachers’ sense of efficacy was positively related to pedagogical innovation, both individually and in teams (Teng et al., 2024).

**Figure 1:**
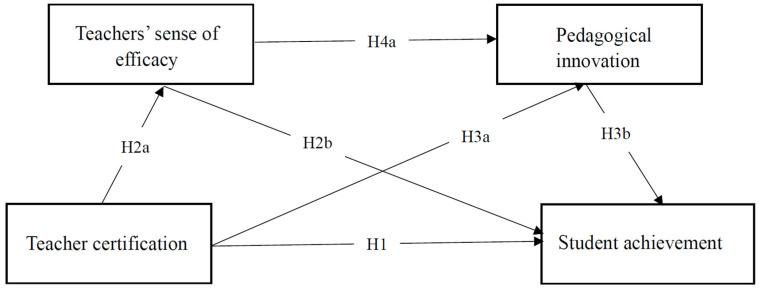
Research Framework.

Based on the above analysis, we develop a research framework, as illustrated in Figure 1.

## 3. Methods

### 3.1. Data

Two datasets are used in this paper. One is the TALIS 2018 Türkiye teacher data, and the other is the PISA 2018 Türkiye student data.

TALIS 2018 Türkiye teacher data were collected in April 2018 (OECD, 2018b). The survey employed a stratified two-stage probability sampling design. The sampling process was as follows: A total of 150 to 200 schools were sampled using systematic random sampling with probability proportional to size from in-scope schools. 15 to 20 teachers were randomly selected within each participating school (Blömeke et al., 2021). The Turkish sample includes 4000 junior high school teachers from 200 junior high schools (OECD, 2018b). In addition, TALIS 2018 data are publicly available and can be downloaded from the official website.

PISA 2018 Türkiye student data were collected from March to July 2018 (OECD, 2018a). PISA 2018 focused on 15-year-old students as its target population. PISA 2018 surveyed student achievement in math, reading, and science. Reading was a major domain in the PISA 2018 wave. The survey employed a stratified two-stage probability sampling design. The sampling process was as follows: at least 150 schools were sampled using systematic random sampling with probability proportional to size from in-scope schools. 42 or 35 students were randomly selected within each participating school (Blömeke et al., 2021). The Turkish sample includes 6890 students from 186 high schools. After removing missing values for the main variables, 5470 observations were included in the final analysis. In addition, PISA 2018 data are publicly available and can be downloaded from the official website.

The survey topics of TALIS 2018 include teachers’ background and qualifications, current work, and job satisfaction. The survey topics of PISA 2018 include students’ backgrounds, daily learning experiences, and classroom and school conditions. The TALIS 2018 and PISA 2018 have been widely used in the international academic community (Wang, 2025; Zhan et al., 2024). Thus, we used two datasets to achieve our research objectives. Schools in some countries and regions participated in both TALIS 2018 and PISA 2018 at the same time. The two datasets for Türkiye were merged using the unique identifier “pisaschoolid” available in the TALIS 2018 (Gil-Izquierdo & Cordero, 2018). This makes it possible for us to analyze the effect of teacher certification on student achievement.

### 3.2. Measures

#### 3.2.1. Dependent Variables

The dependent variable of this paper is student achievement in math, reading, and science. The achievement of each student in each subject is reported with plausible values (PVs). These PVs are reported as having a mean of 500 and a standard deviation of 100 (OECD, 2018a). The first PV was used in the analysis.

#### 3.2.2. Mediating Variables

Since the path analysis can only be used to analyze relationships between observed variables (Muthén & Muthén, 2017), the latent variables (e.g., teachers’ sense of efficacy and pedagogical innovation) need to be operationalized into observed variables.

Following the existing study (Ye & Wang, 2024), each observed variable is typically calculated by first averaging the scores for each item within a scale, and then taking the mean of those item averages. The operationalized process is as follows:

First, six items from the Teachers’ Sense of Efficacy Scale in the TALIS 2018 teacher questionnaire (OECD, 2018b) were selected. For example, get students to believe they can do well in school work (TT3G34A). These items, measured on a four-point Likert scale from 1 (not at all) to 4 (a lot), had demonstrated reliability and validity.^1^ Then, since teacher data from TALIS 2018 cannot directly match student data from PISA 2018, we compute the average score of each item at the school level using Equation (1). In other words, it is assumed that teachers in the same school share the same sense of efficacy. Finally, the mean of the six average scores was calculated and regarded as the mediating observed variable, teachers’ sense of efficacy.

(1) $$\mathrm{efficacyItem}=\frac{option\;1\times 1+option\;2\times 2+option\;3\times 3+option\;4\times 4}{option\;1+option\;2+option\;3+option\;4}$$

where efficacyItem refers to the average of each item. Option 1 represents the number of teachers who chose “not at all”. Option 2 refers to the number of teachers who chose “to some extent”. Option 3 refers to the number of teachers who chose “quite a bit”. Option 4 refers to the number of teachers who chose “a lot”. Actually, Equation (1) is a weighted average that describes the average of each item.

The operationalized process of pedagogical innovation is similar to that of teachers’ sense of efficacy. The only difference is that pedagogical innovation was measured by four items^2^. For example, most teachers in this school strive to develop new ideas for teaching and learning (TT3G32A).

#### 3.2.3. Independent Variables

Teacher certification was an observed variable. It comes from the TALIS 2018 teacher questionnaire, “How did you receive your first teaching certification” (TT3G04). Its responses include: (1) A regular concurrent teacher education or training programme. (2) A regular consecutive teacher education or training. (3) A fast track or specialized teacher education or training program. (4) Education or training in another pedagogical profession. (5) Subject specific education or only. (6) I have no formal qualification related to the subject I am teaching or to any type of pedagogical education. (7) Other. From option (1) to option (6), the entry requirements for teacher certification decrease in difficulty sequentially. Thus, options 1 to 6 were assigned values in descending order from 6 to 1 to characterize the difficulty level of each teacher certification path. Given that the difficulty of the pathway in option 7 cannot be compared to those of other options, and its sample size was very small, we dropped the observations that chose option 7. Ultimately, the independent variable ‘teacher certification’ is defined by the following equation:

(2) $$\mathrm{TeaCert}=\frac{option\;1\times 6+option\;2\times 5+option\;3\times 4+option\;4\times 3+option\;5\times 2+option\;6\times 1}{option\;1+option\;2+option\;3+option\;4+option\;5+option\;6}$$

where option 1 refers to the number of teachers who obtain certification through the pathway it specifies. The same applies to options 2 through 6. Equation (2) is a weighted average that describes the difficulty of entry requirements for each teacher certification pathway.

#### 3.2.4. Controls

This paper controls for a series of covariates: students’ gender (1 = female, 0 = male), students’ social economic status (SES), the proportion of female teachers in a school, average teaching experience, school location (1= rural, 0= urban), and school type (1 = public, 0 = non-public). Teachers’ average years of schooling in a school were coded as follows: 1 = below ISCED 2011 Level 3, 2 = ISCED 2011 Level 3, 3 = ISCED 2011 Level 4, 4 = ISCED 2011 Level 5, 5 = ISCED 2011 Level 6, 6 = ISCED 2011 Level 7, 7 = ISCED 2011 Level 8.

(3) $$\mathrm{TeaEdu}=\frac{\begin{array}{c} \mathrm{below}\;\mathrm{ISCED}\;2011\;\mathrm{Level}\;3\times 1+\mathrm{ISCED}\;2011\;\mathrm{Level}\;3\times 2+\mathrm{ISCED}\;2011\;\mathrm{Level}\;4\times 3+\\ \mathrm{ISCED}\;2011\;\mathrm{Level}\;5\times 4+\mathrm{ISCED}\;2011\;\mathrm{Level}\;6\times 5+\mathrm{ISCED}\;2011\;\mathrm{Level}\;7\times 6+\mathrm{ISCED}\;2011\;\mathrm{Level}\;8\times 7 \end{array}}{\begin{array}{c} \mathrm{below}\;\mathrm{ISCED}\;2011\;\mathrm{Level}\;3+\mathrm{ISCED}\;2011\;\mathrm{Level}\;3+\mathrm{ISCED}\;2011\;\mathrm{Level}\;4+\\ \mathrm{ISCED}\;2011\;\mathrm{Level}\;5+\mathrm{ISCED}\;2011\;\mathrm{Level}\;6+\mathrm{ISCED}\;2011\;\mathrm{Level}\;7+\mathrm{ISCED}\;2011\;\mathrm{Level}\;8 \end{array}}$$

where below ISCED 2011 level 3 refers to the number of teachers with a diploma below ISCED level 3 in a school; ISCED 2011 level 3, to those with ISCED 2011 level 3 diplomas in a school; ISCED 2011 level 4, to those with ISCED 2011 level 4 diplomas in a school; ISCED 2011 level 5, to those with ISCED 2011 level 5 diplomas in a school; ISCED 2011 Level 6, to those with bachelor’s degrees; ISCED 2011 Level 7, to those with master’s degrees; ISCED 2011 Level 8, to those with doctoral degrees. Equation (3) is a weighted average, describing teachers’ average years of schooling in a school.

### 3.3. Data Analysis

We performed data cleaning, common method bias tests, descriptive analysis, and correlation analyses using Stata 17.0. The Model 6 of the PROCESS macro program in SPSS 26.0 was used to examine the mediating role of teachers’ sense of efficacy and pedagogical innovation in the relationship between teacher certification and student achievement (Hayes, 2022). Additionally, this paper used the bias-corrected percentile bootstrap method with 5000 samples to examine the mediating role of teachers’ sense of efficacy and pedagogical innovation in this relationship. If the 95% confidence interval (CI) did not include zero, the coefficient was considered statistically significant, indicating the mediating role of teachers’ sense of efficacy and pedagogical innovation was established.

## 4. Results

### 4.1. Common Methods Bias Test

This paper used Harman’s single-factor test to assess the impact of common method bias. The results indicated that six factors with eigenvalues greater than 1.0 were extracted. The first factor explained only 19.76% of the total variance, which is below the 40% threshold (Harman, 1976). This means that common method bias was not a significant concern in the data.

### 4.2. Descriptive Statistics and Correlation Analysis

Table 1 reports the descriptive and correlation analysis results. Teacher certification was significantly and positively associated with student achievement in math (r = 0.122, *p* < 0.001), reading (r = 0.119, *p* < 0.001), science (r = 0.135, *p* < 0.001), and pedagogical innovation (r = 0.038, *p* < 0.01). Teachers’ sense of efficacy was significantly and positively related to pedagogical innovation (r = 0.304, *p* < 0.001) and student achievement in math (r = 0.118, *p* < 0.001), reading (r = 0.117, *p* < 0.001), and science (r = 0.117, *p* < 0.001). In addition, pedagogical innovation was positively and significantly related to student achievement in math (r = 0.194, *p* < 0.001), reading (r = 0.199, *p* < 0.001), and science (r = 0.192, *p* < 0.001). These findings generally support the research hypothesis.

### 4.3. Hypothesis Test

**Figure 2:**
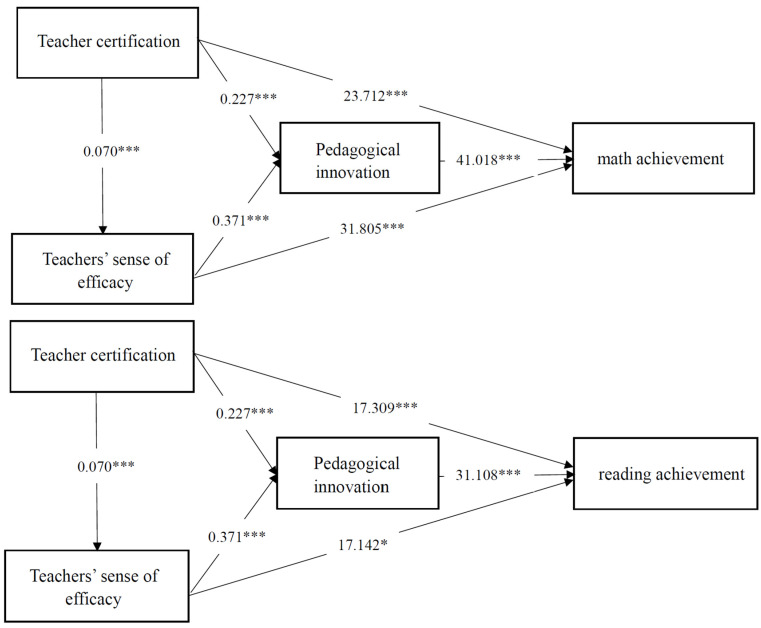

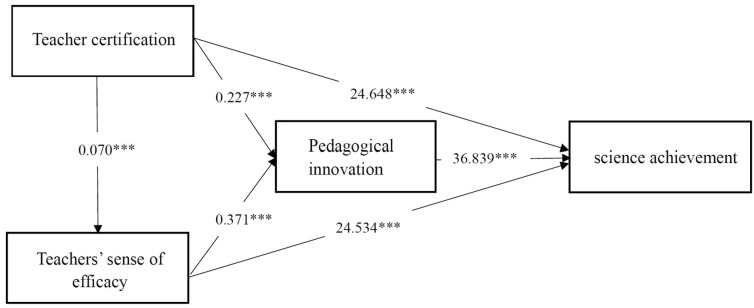
The effect of teacher certification on student achievement in math, reading, and science. * *p* < 0.05, *** *p* < 0.001.

Table 2, Table 3 and Table 4 demonstrate that teacher certification was positively and significantly correlated with student achievement, with significant coefficients for math (β = 36.313, *p* = 0.000), reading (β = 28.143, *p* = 0.000), and science (β = 35.681, *p* = 0.000). Furthermore, teacher certification was also significantly and positively associated with teachers’ sense of efficacy (β = 0.070, *p* = 0.000 across all subjects) and with pedagogical innovation (β = 0.227, *p* = 0.000 across all subjects). Teachers’ sense of efficacy was significantly and positively associated with student achievement, with significant coefficients for math (β = 31.805, *p* = 0.000), reading (β = 17.142, *p* = 0.022), and science (β = 25.534, *p* = 0.001). It was also positively associated with pedagogical innovation (β = 0.371, *p* = 0.000 across all three subjects). Pedagogical innovation was positively and significantly related to student achievement (math: β = 41.018, *p* = 0.000; reading: β = 38.108, *p* = 0.000; science: β = 36.839, *p* = 0.000). Consequently, H1, H2a, H2b, H3a, H3b, H4a were supported. Figure 2 illustrates the path coefficient test results. All hypothetical paths were statistically significant, as the 95% confidence interval did not include zero.

### 4.4. Mediation Effect Test

Table 5 presents the results of the mediation analysis, which was conducted using the bias-corrected percentile bootstrap method. The 95% CI for the direct association did not include zero, indicating that the direct association was statistically significant. Teacher certification was positively correlated with student achievement through direct and three indirect pathways. Direct path: Teacher certification →Student achievement. Indirect path 1: Teacher certification → Teachers’ sense of efficacy →Student achievement. Indirect path 2: Teacher certification → Pedagogical innovation → Student achievement. Indirect path 3: Teacher certification → Teachers’ sense of efficacy → Pedagogical innovation →Student achievement. The coefficient for the direct association of teacher certification with math, reading, and science achievement was 23.712 (95%CI = [11.288, 36.136], *p* < 0.001), 17.309 (95%CI = [5.085, 29.533], *p* < 0.01), and 24.648 (95%CI = [12.837, 36.458], *p* < 0.001), respectively. The coefficients for the indirect association of teacher certification were 12.600 for math (95%CI = [9.933, 15.541], *p* < 0.001), 10.834 for reading (95%CI = [8.353, 13.766], *p* < 0.001), and 11.033 for science (95%CI = [8.580, 13.839], *p* < 0.001). The coefficient for the mediating effect of teachers’ sense of efficacy on math, reading, and science achievement was 2.235 (95%CI = [1.137, 3.846], *p* < 0.001), 1.205 (95%CI = [0.207, 2.530], *p* < 0.001), and 1.724 (95%CI = [0.727, 3.150], *p* < 0.001), respectively. All 95% CI excluded zero. This means that teachers’ sense of efficacy played the mediating role in the relationship between teacher certification and student achievement in math, reading, and science. Similarly, the coefficient for the mediating effect of pedagogical innovation on math, reading, and science achievement was 9.295 (95%CI = [6.979, 11.702], *p* < 0.01), 8.635 (95%CI = [6.374, 11.179], *p* < 0.01), and 8.348 (95%CI = [6.200, 10.694], *p* < 0.01), respectively. All 95% CI excluded zero. This indicates that pedagogical innovation played the mediating role in the relationship between teacher certification and student achievement in math, reading, and science. In addition, the coefficient for the chained mediation effect of teachers’ sense of efficacy and pedagogical innovation on math, reading, and science achievement was 1.070 (95%CI = [0.613, 1.667], *p* < 0.01), 0.994 (95%CI = [0.575, 1.587], *p* < 0.01), and 0.961 (95%CI = [0.541, 1.507], *p* < 0.01), respectively. All 95% CI excluded zero. This means that teachers’ sense of efficacy and pedagogical innovation played the chained-mediating role in the relationship between teacher certification and student achievement in math, reading, and science. To show the relative contribution of each mechanism to total effects, we reported the ratio of each mechanism’s effect size to the total effect size. Results showed that indirect effects accounted for approximately one third of the total effect (math: 34.69%; reading: 38.49%; science: 30.92%). This implies that mechanisms found in this study account for a larger proportion of the relationship between teacher certification and student achievement.

## 5. Conclusions and Discussion

Drawing upon TALIS 2018 Türkiye teacher data, PISA 2018 Türkiye student data, and a path analysis, this paper examines the relationship between teacher certification and student achievement in Türkiye, as well as the mediating role of teachers’ sense of efficacy and pedagogical innovation in this relationship, from the perspective of the PIT.

First, the results demonstrated that teacher certification was positively and significantly associated with high school student achievement in math, reading, and science in Türkiye. This finding is consistent with some previous studies (K. A. Anderson, 2020; Nawas et al., 2025), reinforcing the view that teacher certification can positively predict student achievement. It was found that the effect of teacher certification on student achievement was direct and partially channeled by the two mediators. This finding supports Hypothesis 1. This finding provides us with new insights into the effect of teacher certification. One possible explanation is as follows: In Türkiye, the threshold for teacher certification is relatively higher than in other countries (Aksoy, 2017). Higher thresholds for teacher certification facilitate screening out unqualified and low-quality teachers. In other words, the existing selection mechanism for teacher certification in Türkiye can effectively identify quality teachers who are capable of improving student achievement. Specifically, the content of the teacher certification exam is strongly correlated with the content of teaching work. This alignment, in turn, is directly correlated with student achievement. In addition, improvements in student achievement depend on students’ effort and effective pedagogical strategies. When the threshold for teacher certification rises, a higher proportion of certified teachers have a strong sense of efficacy and innovation. Pedagogical strategies can only play their due role in improving student achievement when teachers with a strong sense of efficacy are able to stimulate students’ enthusiasm for learning. In addition, teachers certified under stringent requirements are more likely to have effective and unique instructional techniques to improve students’ academic performance. Thus, stricter requirements for teacher certification can directly improve student achievement.

Second, it was found that teachers’ sense of efficacy played a partial mediating role in the relationship between teacher certification and student achievement. This means that teacher certification was indirectly correlated with student achievement through teachers’ sense of efficacy. Hypotheses 2a and 2b were confirmed. Our finding is consistent with the PIT. According to this theory, raising the teacher certification threshold leads to improving teacher quality (Goldhaber, 2011). Quality teachers typically have their own unique pedagogical strategies and methods, including stimulating students’ enthusiasm for learning and intrinsic motivation, reducing students’ learning anxiety, updating their pedagogical philosophies in a timely manner, and improving student achievement (Nawas et al., 2025). These advantages undoubtedly enhance their self-efficacy in teaching and educational work. After thresholds for teacher certification rise, candidates who obtain teacher certification must be those with stronger comprehensive competencies, including the ability to implement innovative pedagogy. In this sense, the rise in thresholds for teacher certification contributes to the improvement of teachers’ sense of efficacy. As a key determinant of student achievement, the level of students’ intrinsic motivation has a direct impact (Zhang et al., 2024). Strong enthusiasm for learning relies on teachers’ guidance and encouragement. Specifically, teachers with a strong sense of efficacy set an example for students, uncover students’ strengths, and foster students’ interest in learning. These help students to develop a proper cognitive understanding of learning. Many studies have confirmed the positive relationship between teachers’ sense of efficacy and student achievement using empirical evidence from China (Y. Liu et al., 2022). In conclusion, from the PIT perspective, this paper highlights the critical role of teachers’ sense of efficacy in the process through which teacher certification was positively associated with student achievement, offering new avenues for future research.

Third, the results indicated that pedagogical innovation partially mediated the link between teacher certification and student achievement in the Turkish context. This implies that teacher certification was indirectly associated with student achievement through the identified mechanisms. On the one hand, we found that teacher certification was positively and significantly associated with pedagogical innovation. This finding not only supports Hypothesis 3a but also aligns with the PIT (Maurizi, 1974). After the threshold for teacher certification rises, candidates who can obtain teacher certification are more likely to be qualified teachers. These teachers usually have ambitious career plans. To quickly achieve their career objectives, they have enough incentive to improve their own pedagogical strategies (Jang, 2019) by learning and absorbing frontier pedagogical knowledge, experimenting with new pedagogical practices, and listening to student learning feedback. These actions facilitate updating their pedagogical strategies, leading to improved student achievement. In addition, ambitious teachers with higher intrinsic motivation have subtle yet positive effects on students’ motivation to learn, which in turn facilitates the improvement of student achievement. Thus, as teacher certification thresholds rise, teachers who excel at innovative pedagogy are more likely to be selected. On the other hand, pedagogical innovation positively and significantly predicted student achievement. This finding is consistent with existing studies (Gil-Izquierdo et al., 2023) and supports Hypothesis 3b. Innovative pedagogical strategies contribute to stimulating students’ enthusiasm for learning, help foster their interests, actively increase classroom interaction, and enhance their intrinsic motivation to learn (S. Liu et al., 2024). These actions obviously improve student achievement. In summary, this paper emphasizes the crucial role of pedagogical innovation in the process by which teacher certification was associated with student achievement from the public interest perspective, providing a new avenue for future research.

Finally, the results suggested that teachers’ sense of efficacy and pedagogical innovation served as sequential mediators in the relationship between teacher certification and student achievement in Türkiye. This finding echoes Bandura’s self-efficacy theory (Bandura & Adams, 1977). This theory posits that individual behaviors are shaped by their sense of efficacy. In this study, teachers’ willingness to implement pedagogical innovation is closely linked to their corresponding sense of efficacy. Apparently, teachers with a high sense of efficacy typically have confidence in conducting pedagogical innovation. In addition, teachers’ sense of efficacy was found to be significantly and positively related to pedagogical innovation. Hypothesis 4a is confirmed. According to the PIT, as the threshold for teacher certification rises, the requirements for teachers’ basic skills (e.g., learning and exploring new pedagogical strategies) have also increased accordingly (Goldhaber, 2011). Individuals with high skill requirements are more likely to be competent in their jobs and achieve higher work efficacy. These contribute to implementing innovative pedagogy. Thus, there is a positive relationship between teachers’ sense of efficacy and pedagogical innovation. In addition, an increasing number of studies have shown that teachers’ sense of efficacy positively predicts pedagogical innovation. For example, one study found a positive relationship between teachers’ sense of efficacy and pedagogical innovation in their study using Chinese data (Teng et al., 2024). In addition, teachers’ sense of efficacy and pedagogical innovation not only acted as mediators in the relationship between teacher certification and student achievement, but also exhibited a chain mediation effect. This finding supports Hypothesis 4b. Specifically, teacher certification enhanced student achievement through three indirect paths. The total indirect effect accounted for 34.69%, 38.49%, and 30.92% of the total effect in math, reading, and science, respectively. This indicates the crucial role of teachers’ sense of efficacy and pedagogical innovation in the relationship between teacher certification and student achievement. This finding provides a critical foundation for future research on the relationship between teacher certification and student achievement. This finding provides a critical foundation for future research on the relationship between teacher certification and student achievement.

## 6. Theoretical and Practical Implications

This paper makes the following contributions: First, existing studies only examine the relationship between teacher certification and student achievement (K. A. Anderson, 2020; Cowan & Goldhaber, 2016). This paper reveals the complex mechanisms through which teacher certification was associated with student achievement by focusing on teachers’ sense of efficacy and pedagogical innovation. Second, previous studies have often treated teacher certification as a dichotomous variable to examine the effect of teacher certification on student achievement (Nawas et al., 2025). To the best of our knowledge, this paper is the first to examine the effect of teacher certification on student achievement using a continuous variable measuring the threshold for teacher certification. Third, this paper employed two large-scale OECD datasets, which were merged using a unique school-level identifier (Gil-Izquierdo & Cordero, 2018). This method made full use of information from each dataset and provided a new direction for future research on simultaneously using multiple OECD public datasets. Fourth, although this study is based on Turkish data. Its findings could have some implications for other developing countries because Türkiye is a representative developing country in terms of population and other socio-economic aspects.

Based on the above conclusion, we propose the following policy recommendations. First, this study found an indirect, positive association between teacher certification and student achievement. Therefore, appropriately increasing thresholds for teacher certification could be a pathway to improve student achievement. School management could encourage these teachers, who are certified under high requirements, to share their instructional techniques with other colleagues in order to improve students’ academic achievement. Second, this paper found that teacher certification was associated with student achievement through two mediating variables: teachers’ sense of efficacy and pedagogical innovation. Thus, student achievement improvement was not only positively correlated with the threshold for teacher certification but also related to the enhancement of teachers’ sense of efficacy and the implementation of pedagogical innovation. Third, it was found that teachers’ sense of efficacy directly enhanced student achievement and indirectly did so through its positive association with pedagogical innovation, indicating a chain-mediating effect. Therefore, policymakers should prioritize creating conditions to enhance teachers’ sense of efficacy when increasing the threshold for teacher certification.

## 7. Limitations and Future Directions

There are some limitations that should be kept in mind. First, this paper revealed the correlational relationship between teacher certification and student achievement. Future research could explore the causal relationship in this case. Second, this paper found two mediators between teacher certification and student achievement. Future research could examine other mediators, such as teacher–student relationships and job satisfaction. Third, this study documented the relationship between teacher certification and student achievement. Future studies could examine the relationship between teacher certification and students’ non-cognitive skills, such as achievement motivation and sense of school belonging.

## Figures and Tables

**Table 1:** Descriptive statistics and correlation analysis.

	1	2	3	4	5	6
1. Teacher certification	--					
2. Teachers’ sense of efficacy	0.001	--				
3. Pedagogical innovation	0.038 **	0.304 ***	--			
4. Math achievement	0.122 ***	0.118 ***	0.194 ***	--		
5. Reading achievement	0.119 ***	0.117 ***	0.199 ***	0.803 ***	--	
6. Science achievement	0.135 ***	0.117 ***	0.192 ***	0.833 ***	0.866 ***	--
Mean	5.518	3.248	2.911	453.307	465.837	469.015
SD	0.193	0.145	0.244	86.730	86.129	81.592

Notes: *** *p* < 0.001, ** *p* < 0.01.

**Table 2:** Regression analysis between variables (math).

Outcome Variables	Independent Variables	R	R2	F	β	SE	t	*p*
Math achievement		0.456	0.208	178.922				
	Teacher certification				36.313 ***	6.287	5.776	0.000
	Students’ SES				20.267 ***	0.969	20.915	0.000
	Students’ gender				−2.663	2.128	−1.251	0.211
	Teachers’ diploma				174.529 ***	9.886	17.654	0.000
	Teaching experience				2.403 ***	0.467	5.140	0.000
	Percentage of female teachers				−73.587 ***	7.020	−10.483	0.000
	School location				−24.855 ***	2.340	−10.624	0.000
	School type				−25.018 ***	5.376	−4.653	0.000
Teachers’ sense of efficacy		0.278	0.077	57.049				
	Teacher certification				0.070 ***	0.011	6.190	0.000
	Students’ SES				0.002	0.002	1.009	0.313
	Students’ gender				0.034 ***	0.004	8.476	0.000
	Teacher’s diploma				0.164 ***	0.018	9.164	0.000
	Teaching experience				−0.006 ***	0.001	−7.019	0.000
	Percentage of female teachers				0.044 ***	0.013	3.443	0.001
	School location				−0.015 ***	0.004	−3.528	0.000
	School type				−0.099 ***	0.010	−10.205	0.000
Pedagogical innovation		0.458	0.210	161.070				
	Teacher certification				0.227 ***	0.018	12.772	0.000
	Teachers’ sense of efficacy				0.371 ***	0.021	17.618	0.000
	Students’ SES				0.025 ***	0.003	9.067	0.000
	Students’ gender				0.011	0.006	1.831	0.067
	Teacher’s diploma				0.114 ***	0.028	4.061	0.000
	Teaching experience				−0.021 ***	0.001	−15.956	0.000
	Percentage of female teachers				0.095 ***	0.020	4.815	0.000
	School location				−0.062 ***	0.007	−9.342	0.000
	School type				−0.193 ***	0.015	−12.618	0.000
Math achievement		0.473	0.224	157.520				
	Teacher certification				23.712 ***	6.337	3.742	0.000
	Teachers’ sense of efficacy				31.805 ***	7.624	4.171	0.000
	Pedagogical innovation				41.018 ***	4.763	8.611	0.000
	Students’ SES				19.170 ***	0.966	19.835	0.000
	Students’ gender				−4.696 *	2.122	−2.213	0.027
	Teacher’s diploma				162.166 ***	9.876	16.421	0.000
	Teaching experience				3.546 ***	0.475	7.457	0.000
	Percentage of female teachers				−79.544 ***	6.971	−11.411	0.000
	School location				−21.630 ***	2.337	−9.256	0.000
	School type				−12.457 *	5.450	−2.286	0.022

Notes: * *p* < 0.05, *** *p* < 0.001.

**Table 3:** Regression analysis between variables (reading).

Outcome Variables	Independent Variables	R	R2	F	β	SE	t	*p*
Reading achievement		0.476	0.226	199.749				
	Teacher certification				28.143 ***	6.169	4.562	0.000
	Students’ SES				20.705 ***	0.951	21.775	0.000
	Students’ gender				28.387 ***	2.088	13.595	0.000
	Teachers’ diploma				165.680 ***	9.701	17.079	0.000
	Teaching experience				2.099 ***	0.459	4.575	0.000
	Percentage of female teachers				−40.781 ***	6.888	−5.920	0.000
	School location				−29.951 ***	2.296	−13.046	0.000
	School type				−8.897	5.276	−1.686	0.092
Teachers’ sense of efficacy		0.278	0.077	57.049				
	Teacher certification				0.070 ***	0.011	6.190	0.000
	Students’ SES				0.002	0.002	1.009	0.313
	Students’ gender				0.034 ***	0.004	8.746	0.000
	Teacher’s diploma				0.164 ***	0.018	9.164	0.000
	Teaching experience				−0.006 ***	0.001	−7.019	0.000
	Percentage of female teachers				0.044 ***	0.013	3.443	0.001
	School location				−0.015 ***	0.004	−3.528	0.000
	School type				−0.099 ***	0.010	−10.205	0.000
Pedagogical innovation		0.458	0.210	161.070				
	Teacher certification				0.227 ***	0.018	12.772	0.000
	Teachers’ sense of efficacy				0.371 ***	0.021	17.618	0.000
	Students’ SES				0.025 ***	0.003	9.067	0.000
	Students’ gender				0.011	0.006	1.831	0.067
	Teacher’s diploma				0.114 ***	0.028	4.061	0.000
	Teaching experience				−0.021 ***	0.001	−15.956	0.000
	Percentage of female teachers				0.095 ***	0.020	4.815	0.000
	School location				−0.062 ***	0.007	−9.342	0.000
	School type				−0.193 ***	0.015	−12.618	0.000
Reading achievement		0.488	0.238	170.664				
	Teacher certification				17.309 **	6.235	2.776	0.006
	Teachers’ sense of efficacy				17.142 *	7.502	2.285	0.022
	Pedagogical innovation				38.108 ***	4.687	8.131	0.000
	Students’ SES				19.708 ***	0.951	20.725	0.000
	Students’ gender				26.915 ***	2.087	12.893	0.000
	Teacher’s diploma				156.225 ***	9.717	16.078	0.000
	Teaching experience				3.087 ***	0.468	6.598	0.000
	Percentage of female teachers				−45.773 ***	6.859	−6.674	0.000
	School location				−27.139 ***	2.299	−11.803	0.000
	School type				1.544	5.363	0.288	0.773

Notes: * *p* < 0.05, ** *p* < 0.01, *** *p* < 0.001.

**Table 4:** Regression analysis between variables (science).

Outcome Variables	Independent Variables	R	R2	F	β	SE	t	*p*
Science achievement		0.440	0.194	163.994				
	Teacher certification				35.681 ***	5.966	5.981	0.000
	Students’ SES				18.950 ***	0.920	20.607	0.000
	Students’ gender				11.085 ***	2.019	5.489	0.000
	Teachers’ diploma				156.817 ***	9.382	16.715	0.000
	Teaching experience				1.971 ***	0.444	4.443	0.000
	Percentage of female teachers				−45.355 ***	6.662	−6.808	0.000
	School location				−23.323 ***	2.220	−10.504	0.000
	School type				−10.755 *	5.102	−2.108	0.035
Teachers’ sense of efficacy		0.278	0.077	57.049				
	Teacher certification				0.070 ***	0.011	6.190	0.000
	Students’ SES				0.002	0.002	1.009	0.313
	Students’ gender				0.034 ***	0.004	8.746	0.000
	Teacher’s diploma				0.164 ***	0.018	9.164	0.000
	Teaching experience				−0.006 ***	0.001	−7.019	0.000
	Percentage of female teachers				0.044 ***	0.013	3.443	0.001
	School location				−0.015 ***	0.004	−3.528	0.000
	School type				−0.099 ***	0.010	−10.205	0.000
Pedagogical innovation		0.458	0.210	161.070				
	Teacher certification				0.227 ***	0.018	12.772	0.000
	Teachers’ sense of efficacy				0.371 ***	0.021	17.618	0.000
	Students’ SES				0.025 ***	0.003	9.067	0.000
	Students’ gender				0.011	0.006	1.831	0.067
	Teacher’s diploma				0.114 ***	0.028	4.061	0.000
	Teaching experience				−0.021 ***	0.001	−15.956	0.000
	Percentage of female teachers				0.095 ***	0.020	4.815	0.000
	School location				−0.062 ***	0.007	−9.342	0.000
	School type				−0.193 ***	0.015	−12.618	0.000
Science achievement		0.456	0.208	142.999				
	Teacher certification				24.648 ***	6.024	4.091	0.000
	Teachers’ sense of efficacy				24.534 ***	7.248	3.385	0.001
	Pedagogical innovation				36.839 ***	4.528	8.136	0.000
	Students’ SES				17.972 ***	0.919	19.561	0.000
	Students’ gender				9.394 ***	2.017	4.658	0.000
	Teacher’s diploma				146.374 ***	9.388	15.592	0.000
	Teaching experience				2.973 ***	0.452	6.578	0.000
	Percentage of female teachers				−50.529 ***	6.627	−7.625	0.000
	School location				−20.486 ***	2.222	−9.221	0.000
	School type				0.126	5.181	0.024	0.981

Notes: * *p* < 0.05, *** *p* < 0.001.

**Table 5:** Total, direct, and indirect effects of teacher certification and student achievement.

			Bias-Corrected 95%CI		
Panel A Math	Effect	SE	Lower	Upper	Ratio
Total effect	36.313	6.287	23.988	48.637	
Direct effect	23.712	6.337	11.288	36.136	65.29%^3^
Indirect effect	12.600	1.435	9.933	15.541	34.69%
TC-TS-MH	2.235	0.663	1.137	3.846	6.15%^4^
TC-PI-MH	9.295	1.214	6.979	11.702	25.59%
TC-TS-PI-MH	1.070	0.261	0.613	1.667	2.94%
Panel B Reading					
Total effect	28.143	6.169	16.049	40.237	
Direct effect	17.309	6.235	5.085	29.533	61.50%
Indirect effect	10.834	1.379	8.353	13.766	38.49%
TC-TS-RE	1.205	0.588	0.207	2.530	4.28%
TC-PI-RE	8.635	1.218	6.374	11.179	30.68%
TC-TS-PI-RE	0.994	0.250	0.575	1.587	3.53%
Panel C Science					
Total effect	35.681	5.966	23.985	47.377	
Direct effect	24.648	6.024	12.837	36.458	69.07%
Indirect effect	11.033	1.335	8.580	13.839	30.92%
TC-TS-SC	1.724	0.600	0.727	3.150	4.83%
TC-PI-SC	8.348	1.145	6.200	10.694	23.39%
TC-TS-PI-SC	0.961	0.239	0.541	1.507	2.69%

Note: TC refers to teacher certification. TS refers to teachers’ sense of efficacy. PI refers to pedagogical innovation. MH refers to math achievement. RE refers to reading achievement. SC refers to science achievement.

## Data Availability

The data used in this study are publicly available from the website: https://www.oecd.org/en/data/datasets/talis-2018-database.html.

## Notes

1 The CFA of six items showed a good model fit (χ^2^ = 31.356, df = 6, χ^2^/df = 5.226, CFI = 0.998, TLI = 0.994, RMSEA = 0.033, SRMR = 0.008). The Cronbach’s alpha coefficient for the six items is 0.8597.

2 The CFA of four items showed a good model fit (χ^2^ = 15.468, df = 2, χ^2^/df = 7.734, CFI = 0.998, TLI = 0.994, RMSEA = 0.041, SRMR = 0.006). The Cronbach’s alpha value for the four items is 0.9519.

3 65.29% = 23.712/36.313 × 100%.

4 6.15% = 2.235/36.313 × 100%.
